# Auto-assessment tools for mechanical computer aided design education

**DOI:** 10.1016/j.heliyon.2019.e02622

**Published:** 2019-10-22

**Authors:** Kaur Jaakma, Panu Kiviluoma

**Affiliations:** Department of Mechanical Engineering, School of Engineering, Aalto University, Puumiehenkuja 5, 02150 Espoo, Finland

**Keywords:** Mechanical engineering, Education, Machine design, Computer-aided engineering, Pedagogy, Teaching research, Evaluation methodologies, Improving classroom teaching, STEP, Auto-assessment

## Abstract

Traditionally Computer Aided Design (CAD) courses have been carried out in computer classrooms requiring great amount of teaching personnel. Assessment of students' modeling exercises has been both time consuming and error prone. Utilization of the teaching resources could clearly benefit from online auto-assessment. The auto-assessment tools are widely in use in programming and language courses, but suitable tools for assessing 3D models used in CAD are lacking. This paper presents two new online auto-assessment tools to support the development of both command (“what steps are needed to create this shape?”) and strategic (“how should I model this shape?”) knowledge while learning CAD. The first tool is based on neutral file format (in this case STEP) and can recognize surface differences between student's model and reference model. This tool can assess student's skill to create certain predefined shape (i.e. command knowledge). The second auto-assessment tool utilizes commercial CAD software's API (Application Programming Interface) to test how student's model behaves when modeling parameters are changed. This tool assess student's capabilities to build and design a CAD model's design intent (i.e. strategic knowledge). Developed tools were tested on three mechanical engineering courses. This paper presents both the tools and the feedback received from the students and teachers. Overall, the auto-assessment tools functioned well and feedback from both students and teachers were positive. The most appreciated tool functionality was time and place independent submission and assessment of exercise works. These new tools able focusing teachers' workload from checking the basic exercises to guiding the learning process.

## 1 Introduction

CAD (Computer Aided Design) courses have been a central part of mechanical engineering curriculums for decades (Ye et al., 2004). The focus of the courses varies from learning to use the tools to choosing the most suitable modeling technique, and the CAD part can be a separate entity or it can be integrated to other courses (Kabouridis et al., 2015). Traditional ways of doing courses and also assessing student's learning in CAD courses are (García et al., 2005): practical exercises (mostly weekly), final examination (by modeling a predefined task within time limits) and project work (bigger modeling task individually or in groups). The number of students in CAD courses can be quite high. For example, in our university the intermediate level CAD course have more than 300 students annually. To manage this student amount, automatization of some parts of the CAD education would be beneficial. Auto-assessment of one common exercise type – modeling a predefined geometry – can save teachers' time significantly and allow them to concentrate on helping students in their learning process.

Auto-assessment of exercises is widely used in fields such as programming (Benford et al., 1993; Sherman et al., 2013), language teaching (Yu and Zhang, 2017) and mathematics (Lan et al., 2015; Sangwin and Köcher, 2016). With auto-assessment tools, students can test their knowledge and get almost instant feedback on how well they are progressing.

3D geometry can be assessed by projecting geometry into 2D images from different predefined angles and by using image recognition. This demands a well-predefined template file as well as strict rules about the orientation of the models (Sanna et al., 2012). This method seems to be more suitable for the freehand and artistic kind of modeling, but not for assessing mechanical CAD program's exact geometry creation – the small details are very important in the mechanical engineering.

Besides speeding up the assessment process, auto-assessment tools can be used to ensure the fairness of the student work evaluation. This has been one of the motivators in the develop of auto-assessment tools (Ghosh et al., 2002; Sanna et al., 2012). Utilizing CAD software's API to auto-assess exercises has been proposed by Baxter et al. (2003). Their plan was to use Solidworks' Visual Basic macros to assess parts, assemblies and engineering drawings. Checking how CAD was built using different parameter sets through software's API was also proposed (Kirstukas, 2013). CAD program vendor software can be also utilized in assessing CAD models (Ault and Fraser, 2013).

To assess how the CAD model was created and how the design intent was included, CAD program specific tools are needed. However, besides the plans and ideas, there seems to be a lack of auto-assessment tools for 3D CAD education. That could be a result of the more complex structure of the tasks compared to the well-defined programming or language rules.

Computer using skills can be divided into command knowledge (what tools the program has, how to use them) and strategic knowledge (what methods can be used, what approach to take to solve the task) (Bhavnani et al., 2001). Corresponding terms declarative and procedural knowledge are also used in the literature (Hamade et al., 2007). Maintaining design intent (Iyer and Mills, 2006; Otey et al., 2018) in the modelling process is a part of procedural knowledge. Majority of the educational practices in CAD rely on building up declarative and specific procedural command knowledge (how to use this tool), not on strategic knowledge (Camba et al., 2016).

The native CAD file formats are closed and subject to change with new software versions. However, CAD model geometry can be transferred from one CAD software to another by using either commercial, software specific importers developed by vendors, or neutral file formats, such as STEP or IGES (Kirkwood and Sherwood, 2018; Pratt, Anderson and Ranger, 2005). By using neutral file formats, the auto-assessment method can be used with almost any commercial CAD program, and they also support several existing open source 3D CAD software in the market (Di Angelo et al., 2016). As the neutral file formats mostly only transfer the geometry of the model, the information about how the model was built (feature/model tree, dimensions/parameters) is not transferred. When assessing if the student is able to create a predefined shape, neutral file format is sufficient. When checking the modeling approach and steps taken (i.e. how the model was built, design intent), other methods are needed.

In this paper, two auto-assessment tools are presented:

− Geometry assessment tool; a neutral file format based tool that is able to recognize if the shape of the model is right (i.e. the final output is correct). This can be used to assess students' command knowledge.
− Design Intent assessment tool; a program specific tool that is able, in addition to assessing the right shape, to also modify students' models and thus test what approach is used while building the model (i.e. what tools are used). This can assess students' strategic knowledge.

These tools were tested on three mechanical CAD courses and students' perceptions on these tools were surveyed.

## 2 Methods

### 2.1 Auto-assessment tools

*Geometry assessment* tool is targeted for intermediate level CAD teaching, during which students learn how 3D CAD geometry can be created and what tools the software packages include. The second tool, *Design Intent assessment*, is targeted for students who are already familiar with CAD and are learning the good modeling practices including how the model behaves when dimensions of some features are changed.

#### 2.1.1 Geometry assessment

*Geometry assessment* tool enables checking that the CAD geometry created by student corresponds to the intended geometry, i.e. how well the command knowledge is applied. The neutral file format STEP (ISO, 2016) is used in the process. The STEP format was chosen because of good readability (plain text format) and good export support by both commercial and open source CAD programs.

**Fig. 1:**
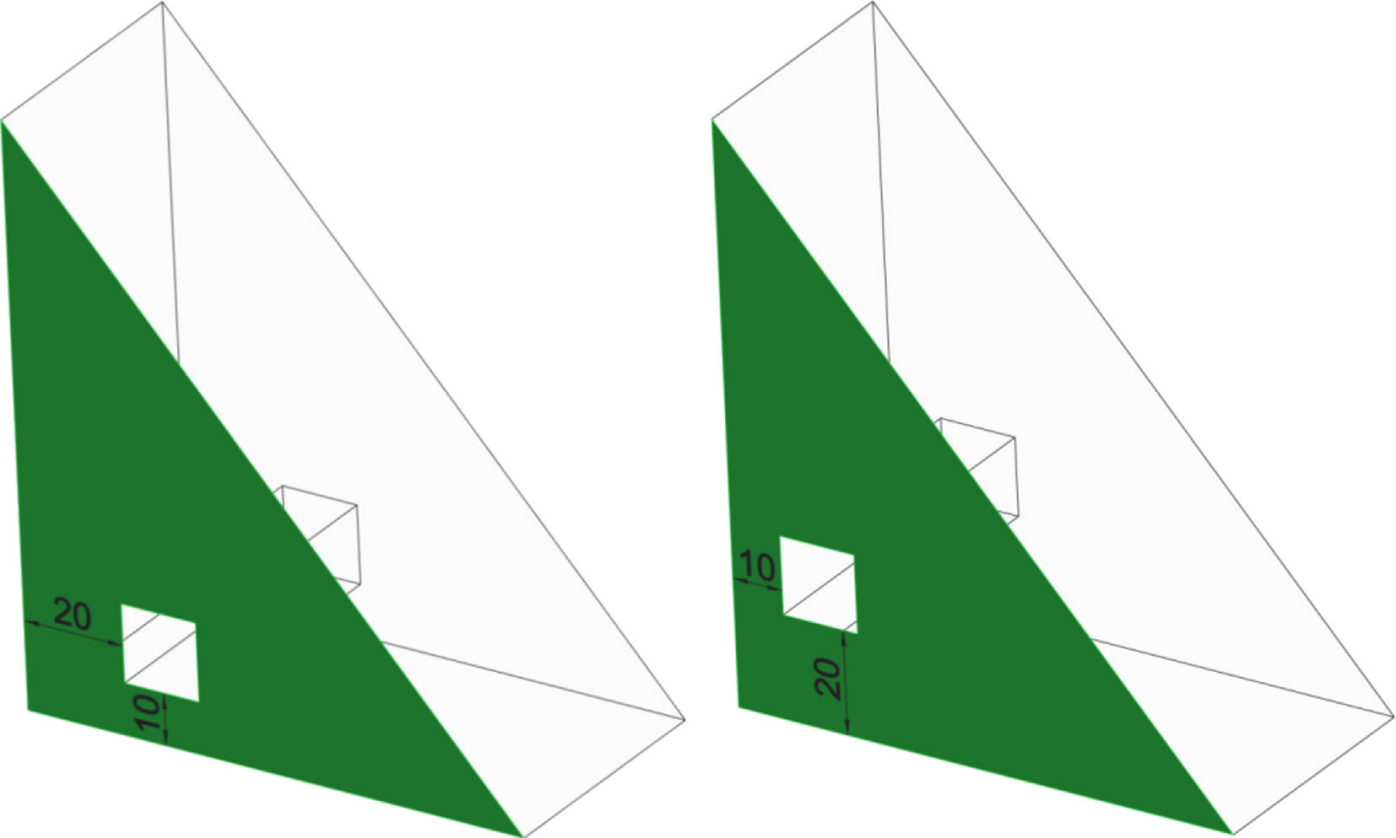
Two models where the highlighted surface has the same circumference but the square cuts have different locations.

The assessment tool, written in C, reads the reference model's surfaces in STEP format and tries to find corresponding surfaces (ADVANCED_FACE in STEP file) from student's STEP file. The similarity is defined by calculating the circumference of a surface. If the surface is not found, it will be marked as wrong in the feedback. In addition to finding the same-sized surfaces, the tool is able to calculate the distance between surfaces. This is an useful tool when the circumference of a surface is same but the features in the surface are in different places (Fig. 1). By using the surface recognition, the tool is not depended on the coordinate system of the model. However, the tool cannot recognize mirrored models. When learning how to use the modeling tools and commands, this is not considered as a big shortcoming.

**Fig. 2:**
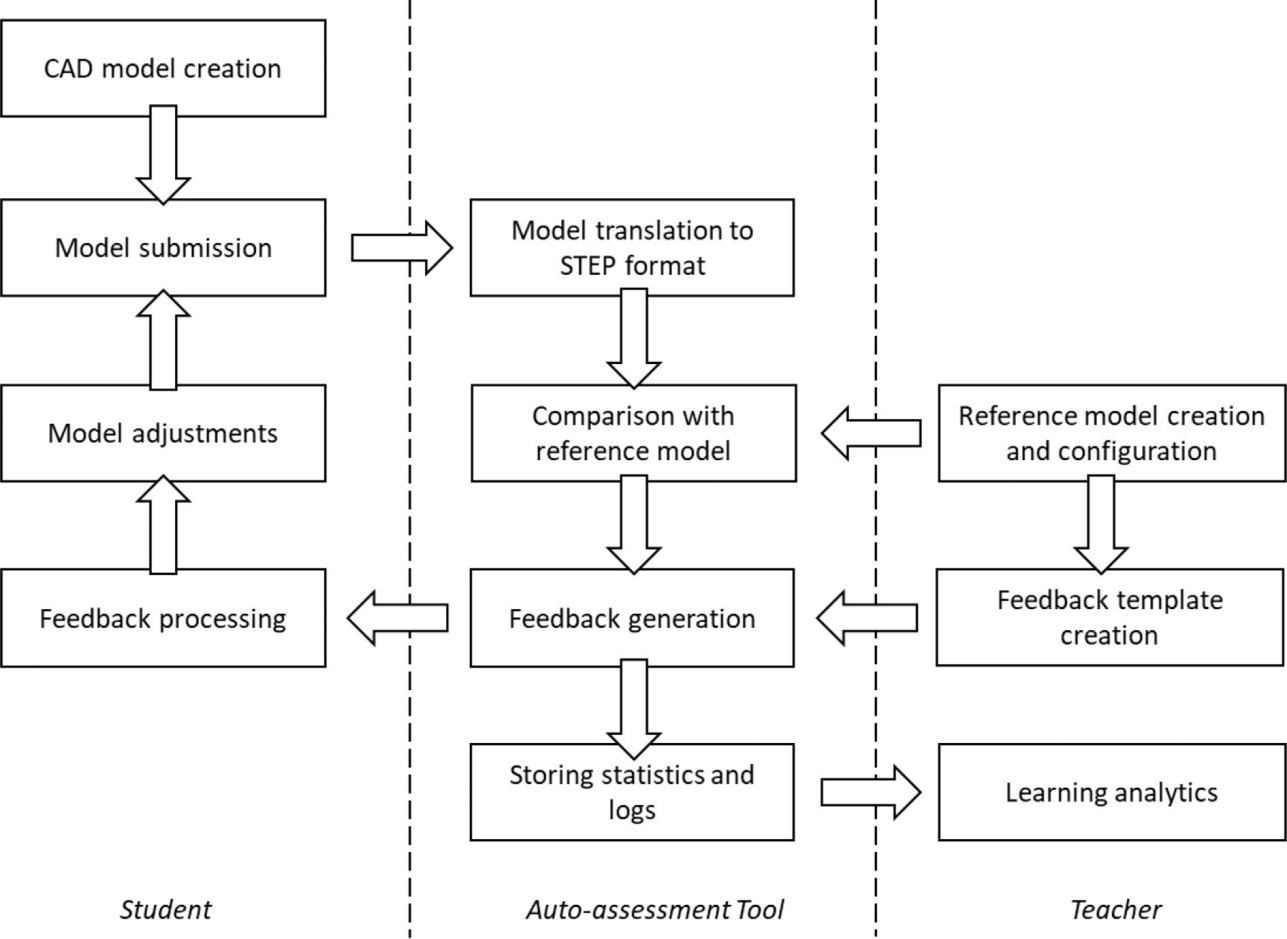
Workflow of geometry assessment tool.

The overall assessment process of the auto-assessment tool is presented in Fig. 2. Student is required to log in through Moodle-based virtual learning environment. In our test case, student used Siemens Solid Edge to create CAD models, and in the server side, Siemens NX was used to export student's models to STEP format to ensure uniformity of files. The students' native CAD-models are stored on the server.

**Fig. 3:**
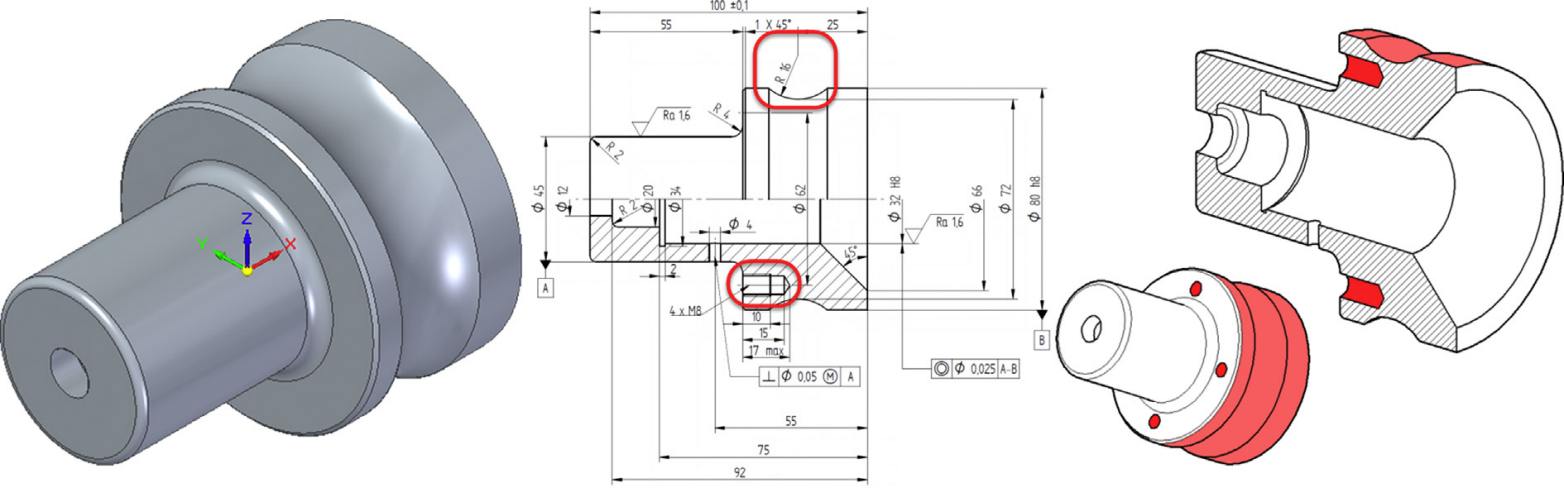
The model assessment steps. In the left: student's model, in the middle: model mistakes highlighted, in the right: auto-assessment tool's feedback highlighting missing features (holes, dark red) and wrong shapes (light red).

The assessment tool provides the students visual feedback. The tool highlights geometries that are missing or have wrong sizes (Fig. 3). The feedback cannot tell which exact feature has wrong shape or dimension, but student has to conclude this based on the hints. For example, the rightmost drawing in Fig. 3 shows that the three outer surfaces have wrong sizes, but not that the mistake was in the groove feature.

**Fig. 4:**
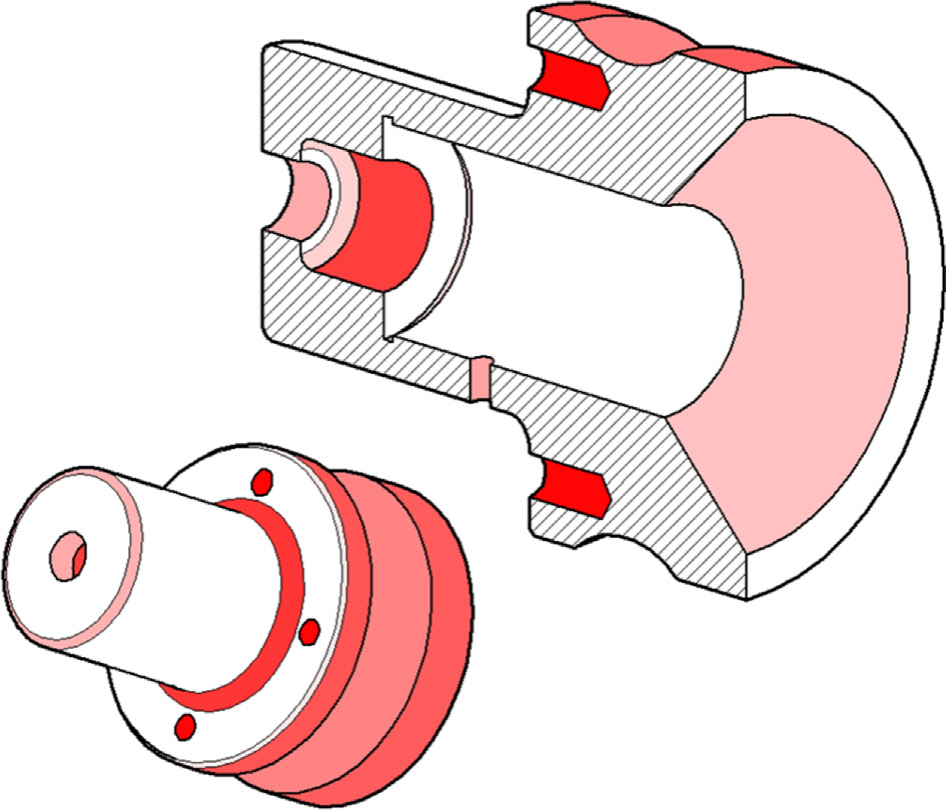
A heatmap of one exercise model. The darker the area, the more mistakes were found.

The auto-assessment tool collects detailed information from all the submissions and presents the most common mistakes by using heatmaps. As seen on an example (Fig. 4), the darker the area is, the more mistakes related to that area were done by the students. This helps teachers to develop the tasks and the guidance, and even to provide direct support to selected students during the course.

#### 2.1.2 Design intent assessment

*Design Intent Assessment* tool enables assessment of strategic knowledge (how should I model this shape?). It can check that the student's model has the right size (surface area and volume), but since it works with native files it also enables testing of the model with additional ways, for example, executing model with different parameter sets (Table 1). This is a very useful option when checking that model's design intent is working as designed, i.e. model behaves right with certain inputs.

**Table 1:** Different parameter sets, corresponding geometry and calculated checker values.

Parameters	Geometry	Surface Area (mm^2^) Volume (mm^3^)
SIZE_H: 4 SIZE_V: 2 THIN: FALSE		3842.4 2375.4
SIZE_H: 2 SIZE_V: 2 THIN: TRUE		1040.2 612.6
SIZE_H: 3 SIZE_V: 1 THIN: FALSE		1711.2 1213.4
SIZE_H: 1 SIZE_V: 2 THIN: FALSE		1172.3 829.9
SIZE_H: 1 SIZE_V: 1 THIN: TRUE		305.8 184.3

**Fig. 5:**
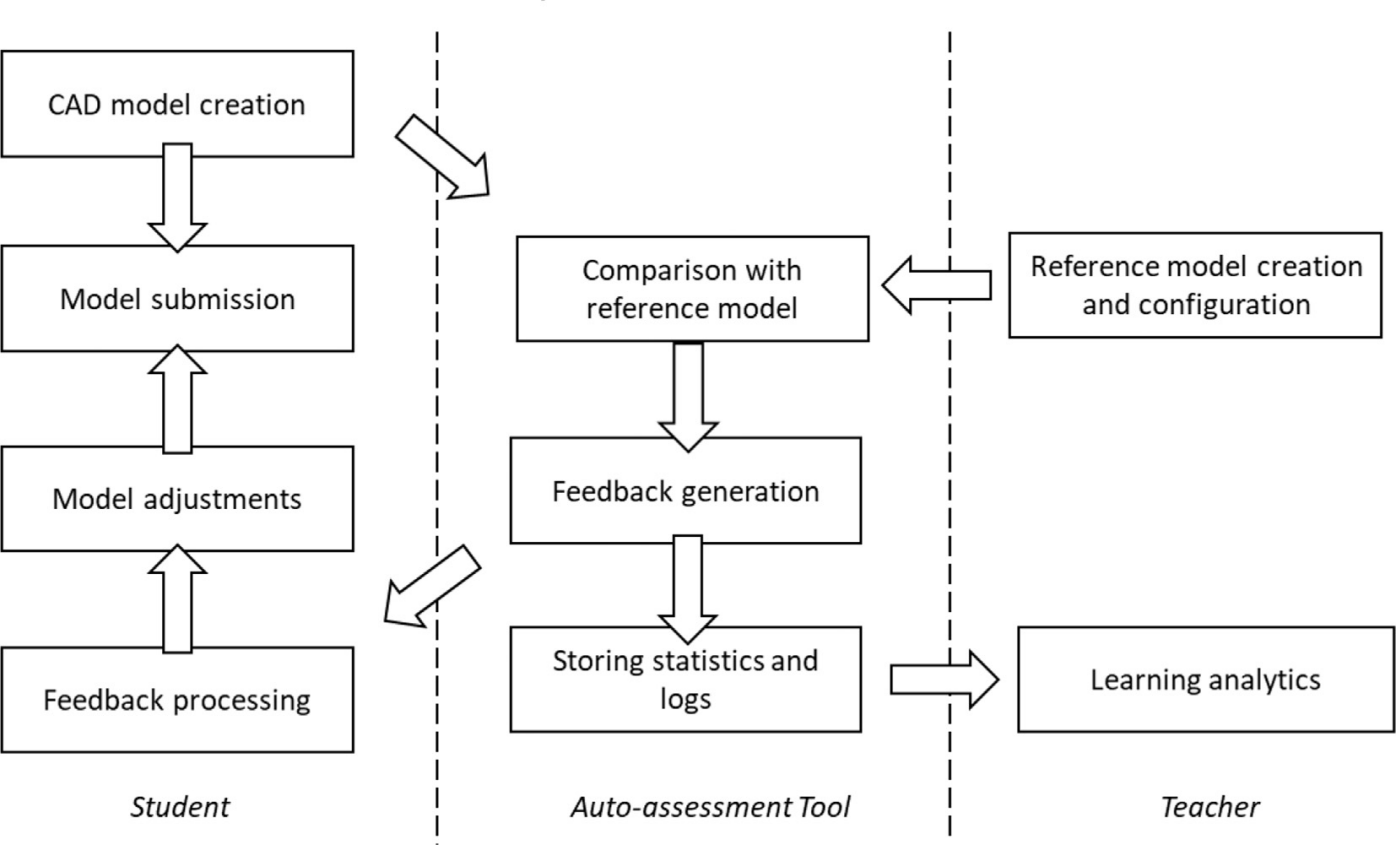
Workflow of design intent assessment tool.

The assessment process of this auto-assessment tool is presented in Fig. 5. The tool requires students to log in through our virtual learning environment (Moodle-based). The auto-assessment tool is built on Windows Server 2016 and contains Django Web framework for user interface, PTC Creo Parametric 3.0 M110 to open and run CAD models, Python (version 3.5.4) wrapper for Creo's Visual Basic API for commanding Creo, and Celery for queue tasks. The same tests are run for both student's and reference models utilizing Creo's API.

Several tests are integrated to the tool:

− MassPropChecker, allows calculation of mass properties, such as volume or surface area.
− RegenChecker, allows testing if model regenerates with given values.
− ModelTreeChecker, allows testing if the model has certain features.

**Fig. 6:**
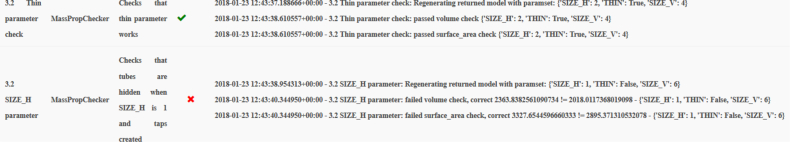
A part of summary table showing what checks were run and what the result was.

The tool gives the user feedback on what kind of tests were run, what parameter values were checked and what was the result. If the test fails, the tool tells the expected value (surface area or volume) to the student by presenting a summary table (Fig. 6). In the figure, a part of summary table is presented showing that returned model does not work when one parameter (SIZE_H) of the part is 1. Unlike in *Geometry Assessment* tool, there is no visual feedback on the model. The idea is that student tests the model with the same parameter values as the auto-assessment tool had and figures out the reason for the misbehavior in design intent.

### 2.2 Student surveys

During fall 2017, both auto-assessment tools were piloted on three different CAD related courses (Table 2). The same online survey was carried out for each course after the obligatory CAD exercises were completed. The permission to use students' answers in the research was asked and the study followed our university's ethical guidelines^1^. In addition, the answers were anonymized. The students were asked to give feedback on the tool (was it easy, how much assistance was needed etc.) and how it could be developed further. In addition, attitudes toward auto-assessment (do you trust that tool test correctly etc.) and opportunity to complete modeling task at home were surveyed.

**Table 2:** CAD related courses used in auto-assessment tool testing.

Course Name	Auto-assessment Tool	Amount of students in test group	Amount of auto-assessed exercises
Engineering Design Basics A	Geometry	75	1 (9 variations)
Engineering Design Basics B	Geometry	39	3
Machine Design	Design Intent	92	1

*Geometry Assessment* tool was tested on two mechanical engineering courses. Both courses utilize CAD programs to create 3D geometries. The auto-assessment tool was tested on a selection of modeling exercises with predefined outcomes.

**Fig. 7:**
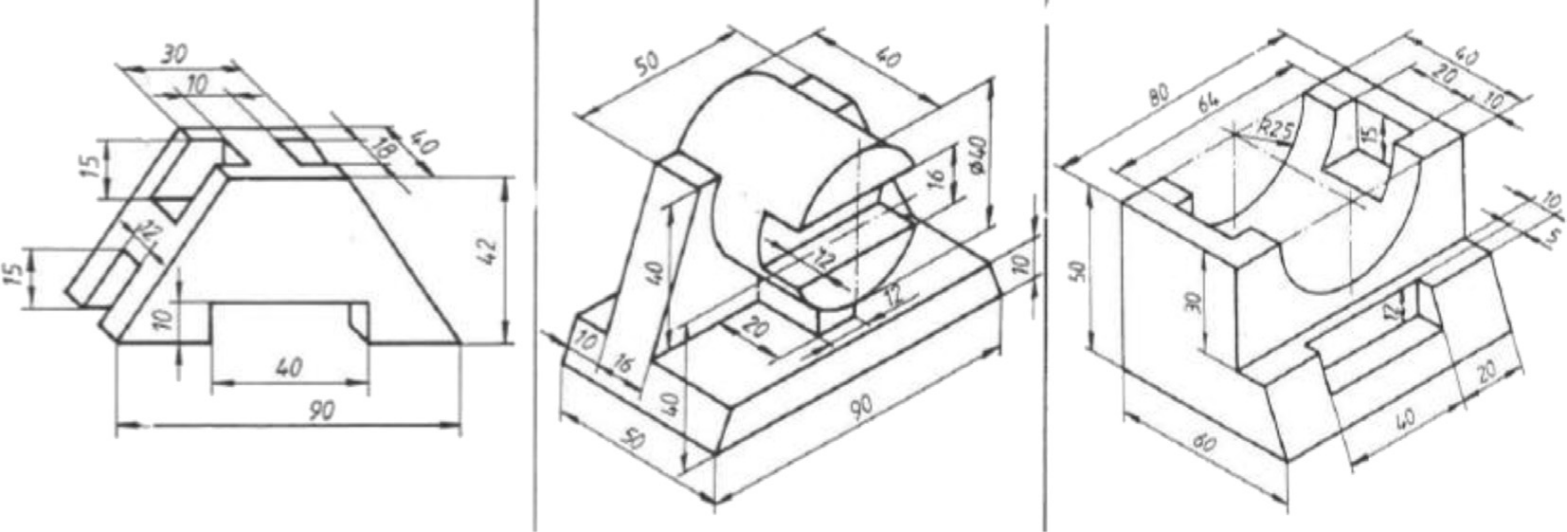
Three example parts out of nine used in the first exercise.

The first course is *Engineering Design Basics A*, a third year bachelor level course about engineering drawings, standards and design for manufacturing. Annually about 90 students take this 5 ECTS, course. The course is aimed for students in mechanical and civil engineering major, and they already have basic CAD knowledge from previous courses. The auto-assessment tool was used in one obligatory weekly modeling exercise, where a 3D model based on a drawing had to be created. There were nine different models to choose from (Fig. 7).

**Fig. 8:**
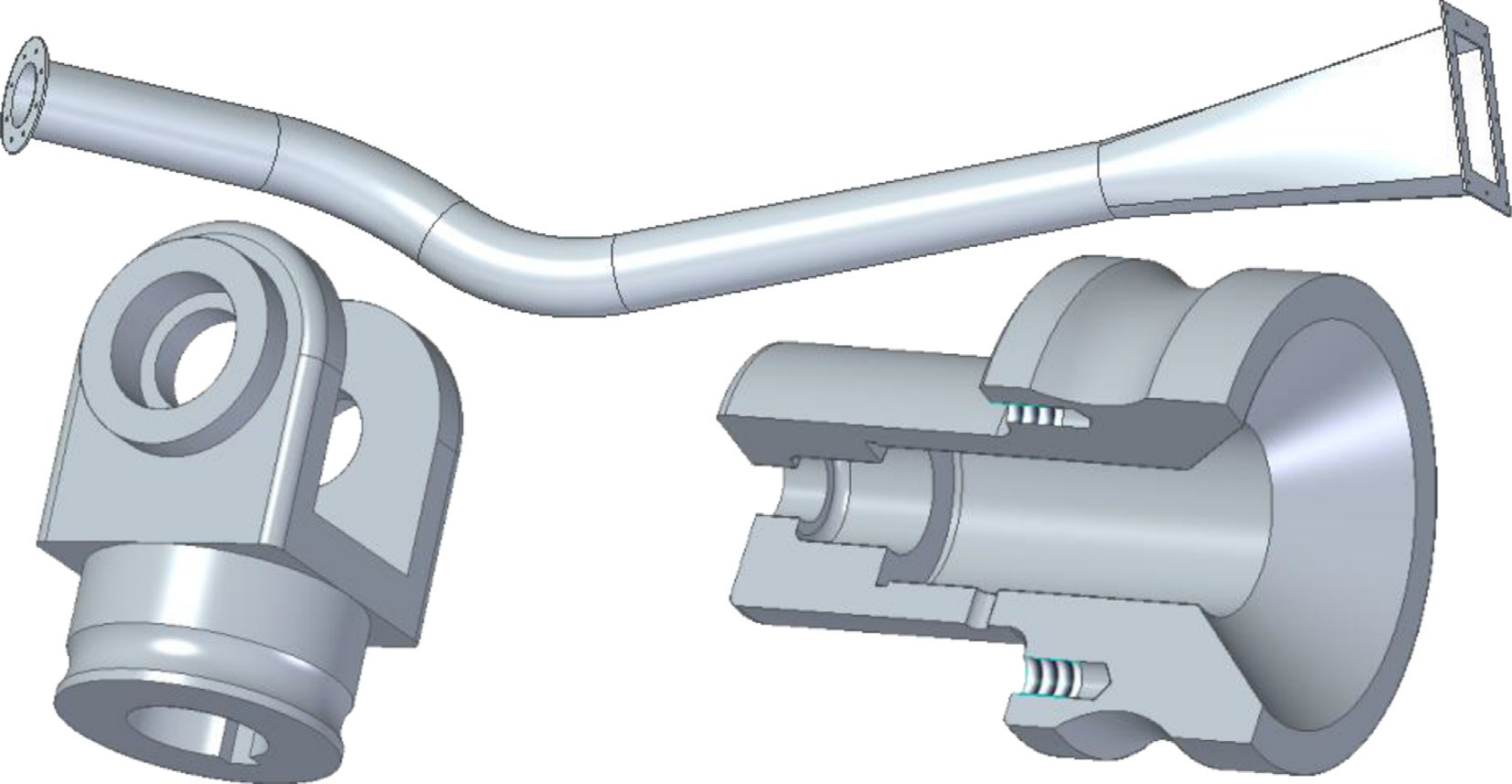
Three auto-assessed models done during the course (not in scale).

The second course is *Engineering Design Basics B*, a second year bachelor level course about CAD, engineering drawings, mechanical engineering and machine element design. Annually about 60 students take this 5 ECTS course. This course is aimed for students who have no previous CAD expertise. The auto-assessment tool was tested on three exercises (Fig. 8), with pre-defined outcomes.

**Fig. 9:**
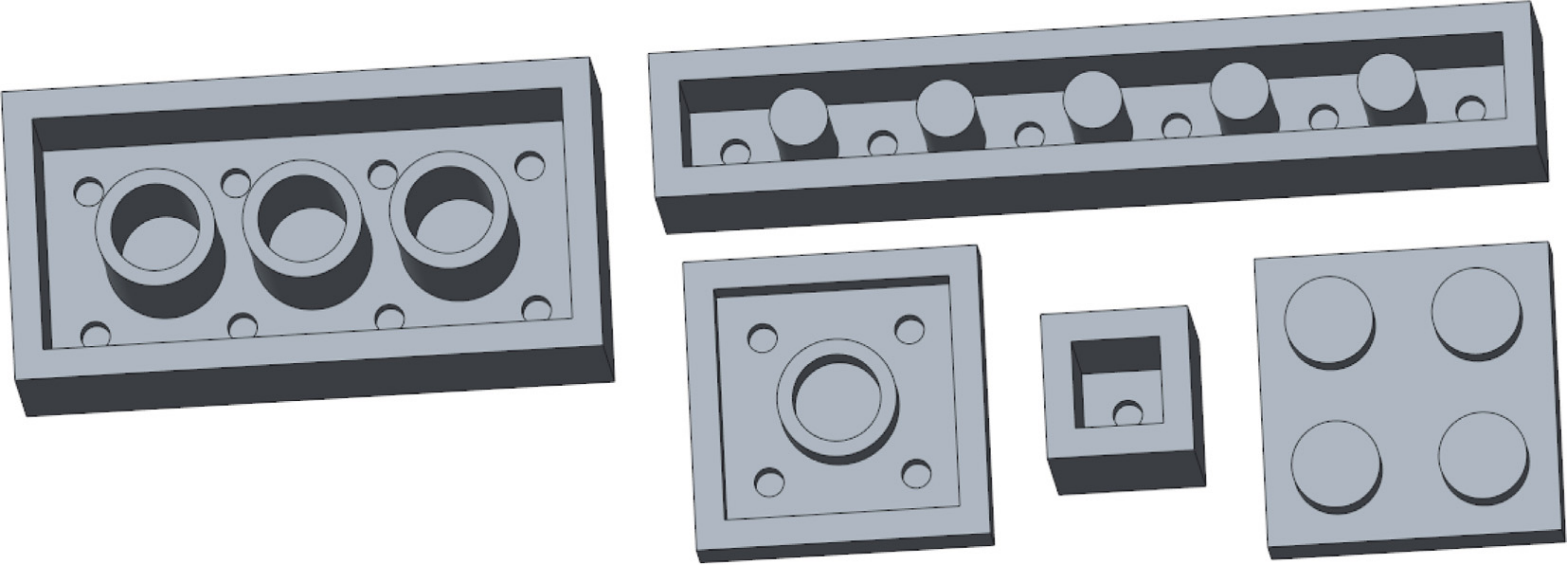
Test model and its different variations.

The *Design Intent Assessment* tool was tested on *Machine Design* course, a first year and 5 ECTS master level course, which has about 100 students annually. The course is about simulation based machine design, where different simulation tools for motion and strength analysis are introduced. The different geometries (with different inputs) of test model can be seen in Fig. 9. Traditionally this course does not have a CAD modeling task.

## 3 Results

In this chapter, combined results from *Geometry Assessment* (N = 114) and *Design Intent Assessment* (N = 92) auto-assessment tool surveys are presented. In addition, results from different test runs utilizing previous year's student works are presented.

### 3.1 Development tests

During the development of *Geometry Assessment* (GA) tool, a series of tests were run to discover how well the tool can assess models and to check that the returned models were correctly done. The tests were run on two different kind of models: a model done by basic features (protrusion, revolute, hole etc.), and a more complex model containing tangential curves and swept geometries. The instructions were different. For the first model, only an engineering 2D drawing was given. For the second model, a step-by-step video was given. The first model had a mistake rate of 42 out of 127 (33%) and the second one 17 out of 64 (27%).

*Design Intent Assessment* (DIA) tool was tested manually by returning and analyzing student models (N = 95) from previous year's CAD introductory course. These models were collected from course's network drive, where students were asked to backup their models after presenting their model to teaching assistant. It was assumed that those models were approved by teaching stuff, i.e. done right. Based on the metadata of the models, 21.1% of the students did their models with their own computer, 11.6% started modeling with the home computer and completed it on the school computer, and 67.4% did their models with the school computer. 15.8% of the models did not pass the auto-assessment due mistakes presented in Table 3.

**Table 3:** Mistakes in previously returned models. See Table 1 for reference.

Mistake type	N	% of not passed models
no/wrong named parameters	2	13,3 %
basic dimensions wrong	4	26,7 %
THIN parameter wrong	4	26,7 %
SIZE_H parameter wrong	11	73,3 %
SIZE_V parameter wrong	8	53,3 %
1-1-1 case wrong	10	66,7 %

### 3.2 Course tests and surveys

**Fig. 10:**
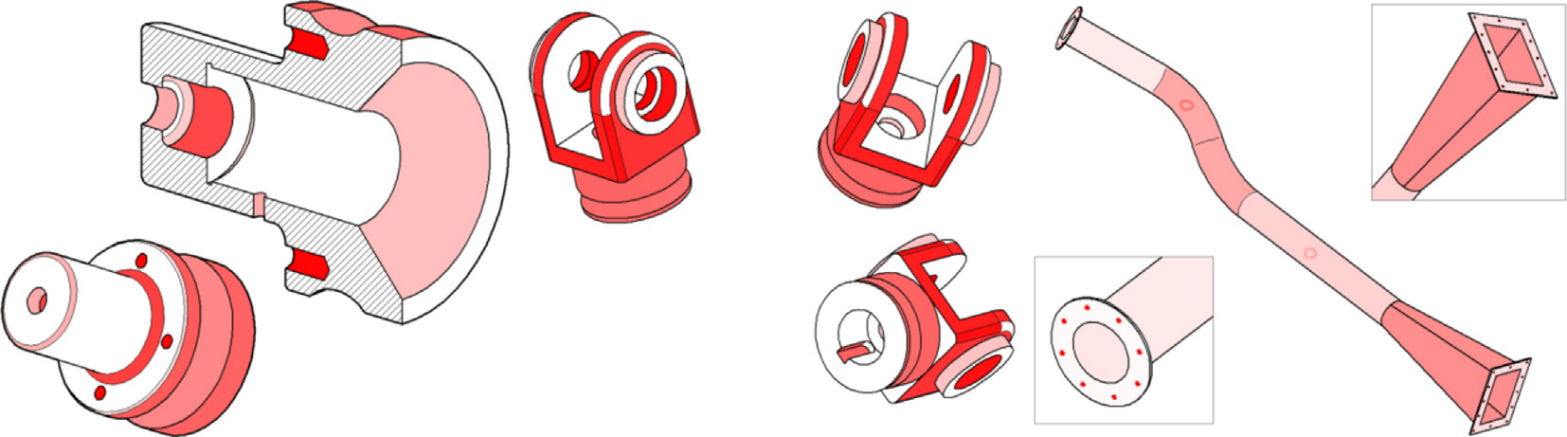
Heatmaps of the mistakes found. The darker the color, the more mistakes.

The GA tool heatmaps from three auto-assessed exercises in *Engineering Design Basics B* course (Fig. 6) are presented in Fig. 10. The number of students who passed exercises are 52, 51 and 46 repeatedly. A student needed an average of nine returns to the tool to pass all obligatory exercises.

Minority of the students (38.6% in *Geometry Assessment* and 39.1% in *Design Intent Assessment* tool survey) had some previous experience with automatic assessment tools, mostly in programming courses (C or Python) or mathematics courses (using STACK etc.).

**Fig. 11:**
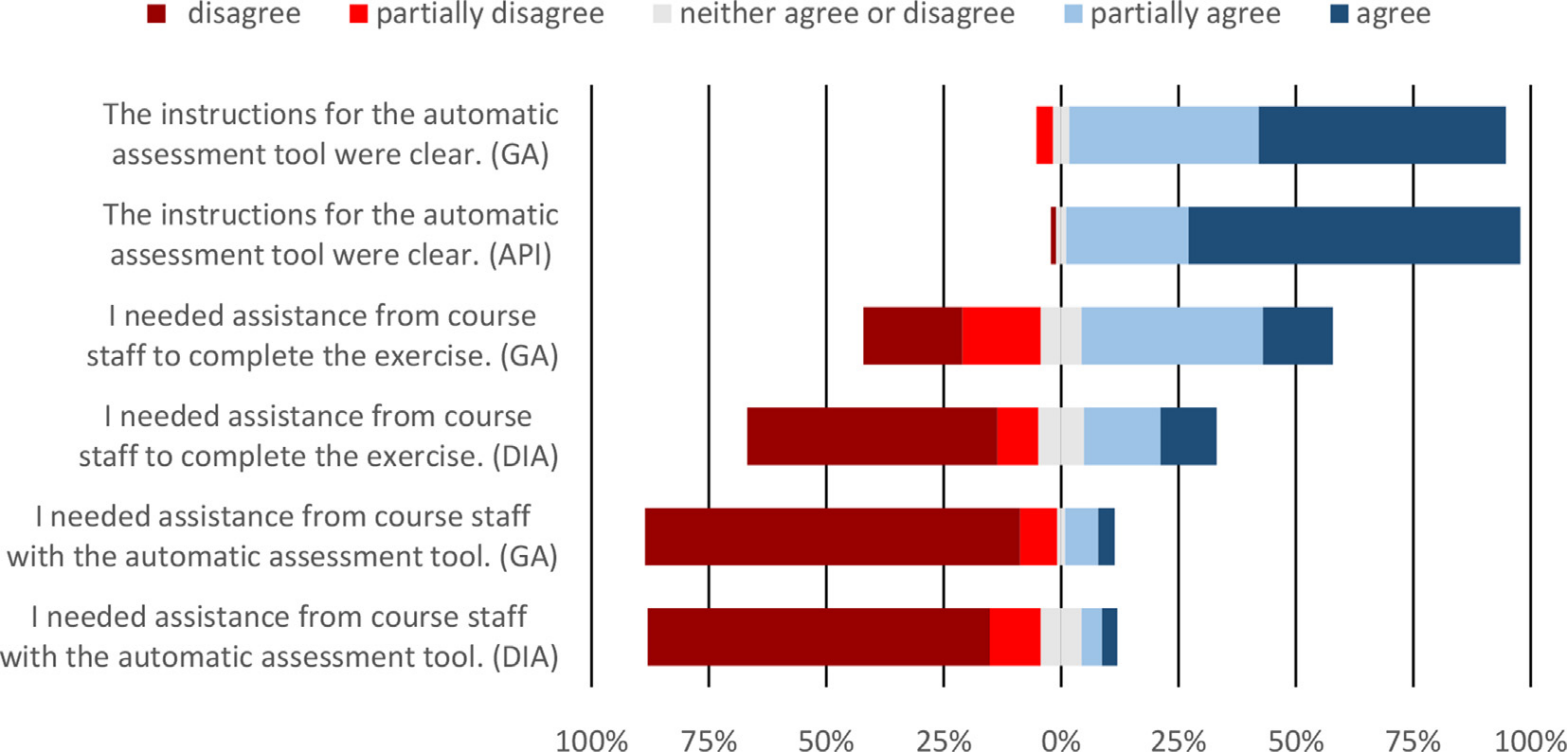
Students' perceptions about instructions and assistance during the exercises.

Minority of the students returned their models at home (31.6% in GA and 17.4% in DIA survey). They needed in average 1.9 (in GA) and 2.2 (in DIA) returns to pass the auto-assessment. When asked how they feel about the possibility of returning their CAD models time and place independent, one student out of 206 did not like the time-independent returning and two did not like the place-independent submitting. Students' perceptions about instructions and assistance during the exercises are shown in Fig. 11.

**Fig. 12:**
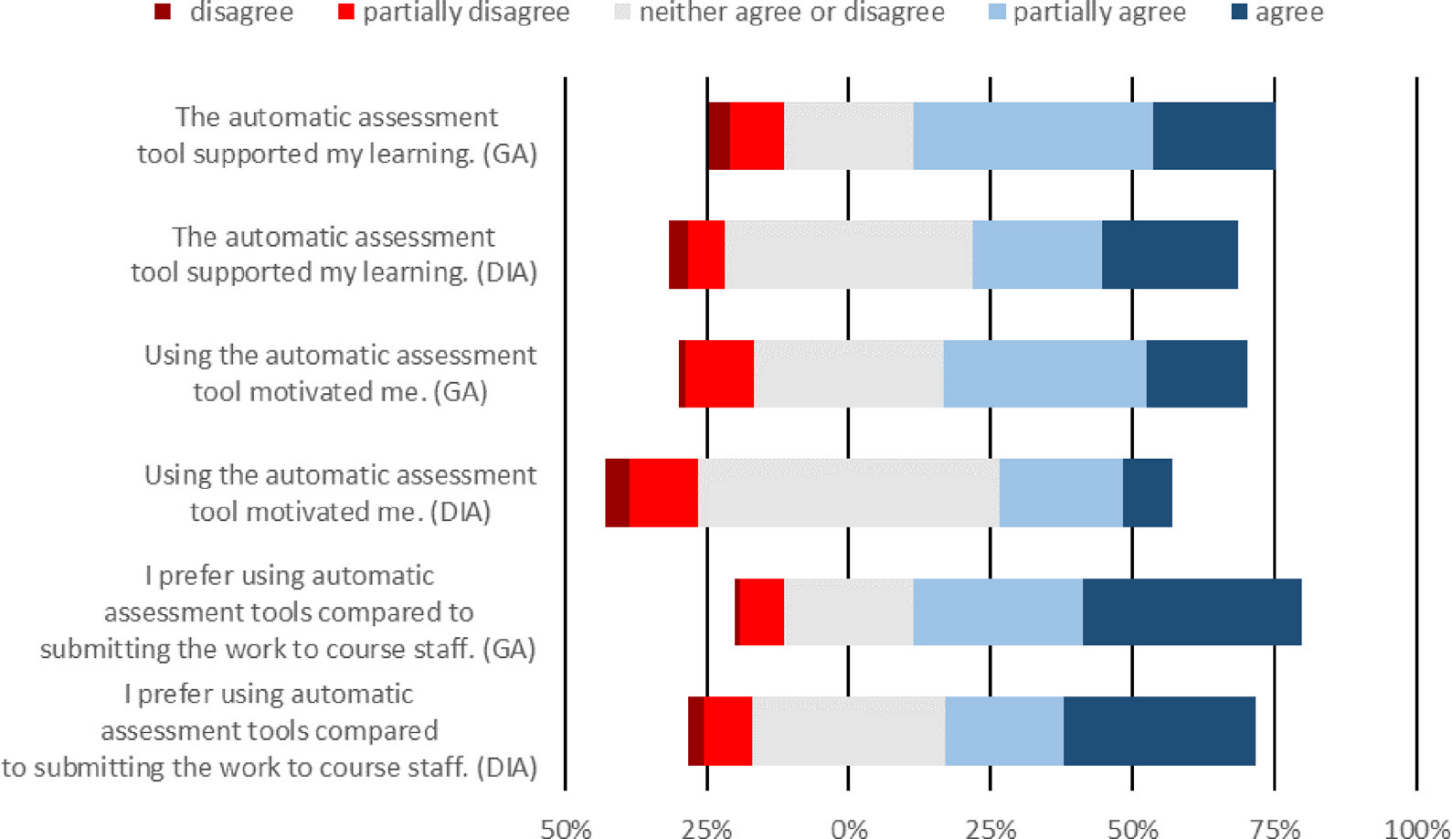
Students' perceptions about how auto-assessment tool's learning experience.

The automatic-assessment tools were found supportive to the learning process (Fig. 12). There were more students who answered “neither agree or disagree” in DIA tool testing group than in GA group.

**Fig. 13:**
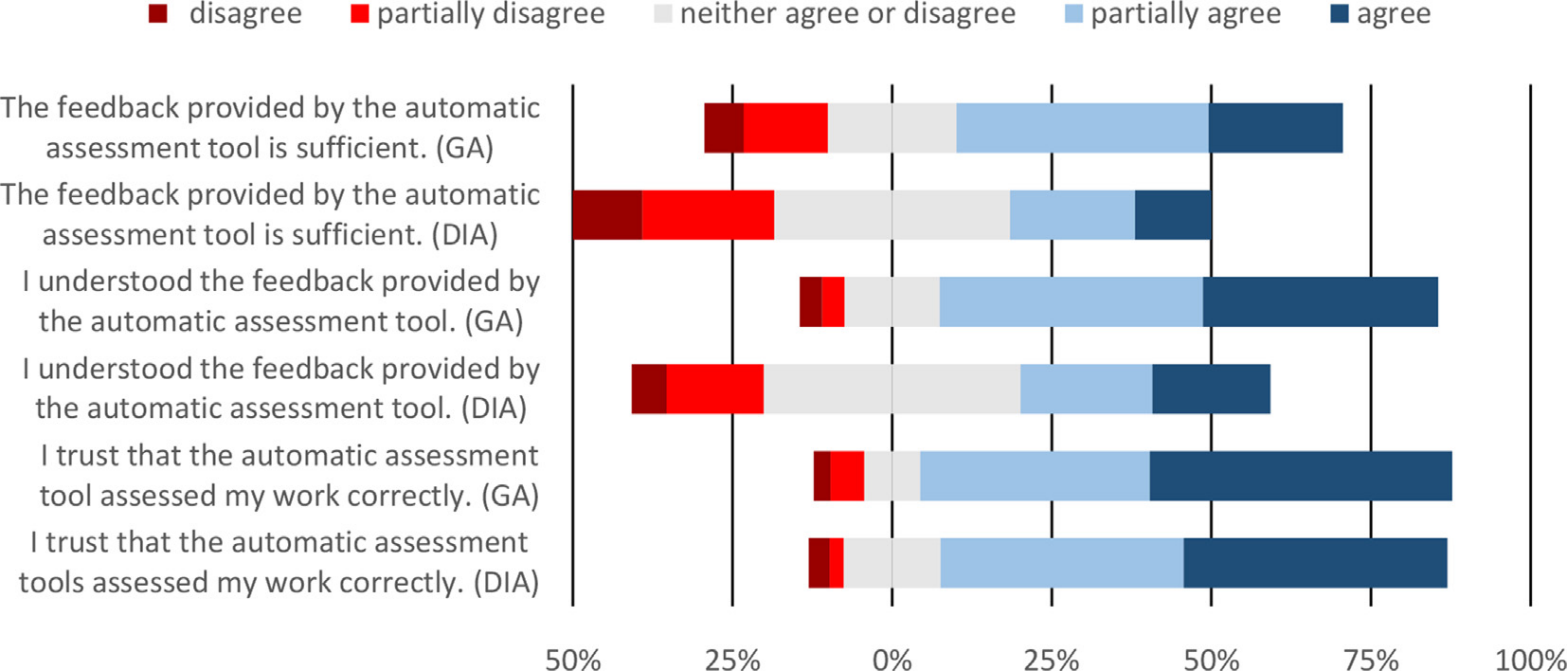
Students' perceptions about feedback provided.

The feedback provided by auto-assessment tools was sufficient (Fig. 13) and the majority of the students (83.3% in GA and 79.3% in DIA) trusted that the automatic assessment tool assessed their work correctly.

In addition to the common questions in the auto-assessment survey, the GA tool survey asked preferred feedback methods (Table 4). The scale was from 1 (not preferred) to 10 (most preferred).

**Table 4:** Preferred feedback types.

Type	AVG	STDEV.P
Right/Wrong	7.8	2.7
Text-based feedback (i.e. “Main hole has wrong dimensions”)	9.1	1.5
Illustrated feedback (wrong features/elements highlighted)	9.4	1.1
Showing the wrongly made feature	9.1	1.5
Giving instructions how the error can be fixed	7.8	2.5

In the free text comments about user experience, the automatic assessment received positive feedback on the possibility to submit works anytime and anywhere. It was also seen useful that before creating engineering drawings, student can check that his/her model was correct. The negative feedback on was about the quality of feedback – the teacher was preferred.

When asked students' comments about learning experience, the feedback type (showing wrong sized surfaces) was seen a little unclear. Some students had a fear that by utilizing auto-assessment, we may reduce the amount of teaching assistants – the tool cannot advice in the using of software and sometimes feedback from a human is needed. Auto-assessment was also seen as an equal assessment method, where differences between teachers' assessments were excluded.

## 4 Discussion

The pilot tests demonstrated that auto-assessment of 3D CAD model is possible and students are willing to use them. Based on the authors' experience, the instruction during the computer classes improved and the sessions ran smoother. A surprising outcome was the increased workload of teacher-in-charge. When student's auto-assessment did not go through when working at home or after consulting computer class sessions, solving the most challenging cases was a task for responsible teacher. Luckily, after solving some of the challenging cases, a pattern was recognized and in the future solving these cases will be smoother.

Analyses done with the models from previous years showed that teachers might miss some modeling errors when assessing student's models. Auto-assessment can both improve the quality of students work (it is not possible to pass the exercises anymore due insufficient checking) and bring equality to grading process (all have exactly the same criteria, no human effect). On the other hand, auto-assessment is limited to models that fulfill predefined criteria and thus can not be used to assess open-ended exercises.

Auto-assessment tools received positive feedback from the students. The students appreciate the possibility to submit their models and get instant feedback time and place independent. Since traditionally models were assessed during the CAD exercise sessions where guidance was also available, there was some fear that the amount of sessions may be reduced. The utilization of auto-assessment tools was planned to handle rather tedious manual checking of predefined models, so now the teachers can concentrate on helping and guiding students during the sessions, if students need it. The modeling tasks and teaching material is designed so that working at home is also possible. Of course, bigger and more creative exercises, where outcome is not strictly predefined, will still be demonstrated personally to the teaching staff.

There was some mistrust among some of the students about the correctness of assessment - they felt that the tool did not assess their work correctly. It appeared that some students returned exactly the same models within short time window. While this does not create a lot of burden to the auto-assessment tools, the tool should recognize the exact same model (by using for example file hash) and prevent returning it again.

When comparing the two tested auto-assessment tools, *Geometric Assessment* tool got better feedback than *Design Intent Assessment* tool. The biggest difference was in the feedback type, which is understandable. The *Design Intent Assessment* tool only provides written feedback, while the feedback of geometric assessment tool is mostly visual. In addition, *Design Intent Assessment* tool was tested on a course that does not have any other obligatory CAD modelling exercises and is also using another CAD environment. This can be seen as a notable difference in amount of assistance from the teaching staff that was needed to complete the exercise.

Auto-assessment tools enable to change the way the CAD will be taught. Traditionally, introductory level courses have used rather complex tasks of creating a model using several commands and steps. In this way, there are several possibilities to make mistakes that are carried out to the next step. With *Geometric Assessment* tool, these rather long modeling tasks can be divided into smaller ones where a certain command or tool can be learned. Previously utilizing these smaller tasks had required small group sizes and many teaching assistants, which with the current number of students are not place or personnel efficient. More emphasis can be put on building up students' strategic knowledge. With *Design Intent Assessment* tool different modeling approaches can be presented and taught. For example, tasks can include a creation of same shaped model by using different modeling approaches.

The future work will include increasing the quality of auto-assessment tools' feedback. The tools should give more accurate hints on how the returned model should be corrected to pass the next return. The *Design Intent Assessment* tool should have some visual feedback on what different tests did and what they tested, and then some additional information how the model should be improved. The feedback of the *Geometry Assessment* tool was good, but there is also need for more intelligent feedback, perhaps by recognizing the most complex errors and by giving targeted guides on how to correct those. The developed tools also enable the development of online education. By offering a completely online course, we can easily provide a structured way to learn mechanical CAD time and place independent for students from different fields.

## 5 Conclusions

This paper presented two auto-assessment tools suitable for mechanical CAD model assessment: a *Geometric Assessment* tool and *Design Intent Assessment* tool. The first tool can assess that the modeled geometry corresponds with the predefined outcome, while the latter assess that the model behaves correctly when the main dimensions are changed. These tools were piloted in three different mechanical engineering courses related to CAD education. The tools worked well during the pilots and received a positive feedback from the students. The work will continue to improve these tools and to create an online course.

## Declarations

### Author contribution statement

Kaur Jaakma: Conceived and designed the experiments; Performed the experiments; Analyzed and interpreted the data; Contributed reagents, materials, analysis tools or data; Wrote the paper.

Panu Kiviluoma: Conceived and designed the experiments; Contributed reagents, materials, analysis tools or data; Wrote the paper.

### Funding statement

This research did not receive any specific grant from funding agencies in the public, commercial, or not-for-profit sectors.

### Competing interest statement

The authors declare no conflict of interest.

### Additional information

No additional information is available for this paper.

## Footnotes

1 https://www.aalto.fi/en/services/research-ethics-and-research-integrity.
